# Role of ferroptosis in radiation-induced soft tissue injury

**DOI:** 10.1038/s41420-024-02003-5

**Published:** 2024-07-05

**Authors:** Charlotte E. Berry, Carter B. Kendig, Nicholas An, Alexander Z. Fazilat, Andrew A. Churukian, Michelle Griffin, Phoebe M. Pan, Michael T. Longaker, Scott J. Dixon, Derrick C. Wan

**Affiliations:** 1 Hagey Laboratory for Pediatric Regenerative Medicine, Division of Plastic and Reconstructive Surgery, Department of Surgery, Stanford University School of Medicine, Stanford, CA USA; 2 Institute for Stem Cell Biology and Regenerative Medicine, Stanford University, Stanford, CA USA; 3 Department of Biology, Stanford University, Stanford, CA USA

**Keywords:** Apoptosis, Target validation, Chronic inflammation

## Abstract

Ionizing radiation has been pivotal in cancer therapy since its discovery. Despite its therapeutic benefits, IR causes significant acute and chronic complications due to DNA damage and the generation of reactive oxygen species, which harm nucleic acids, lipids, and proteins. While cancer cells are more vulnerable to ionizing radiation due to their inefficiency in repairing damage, healthy cells in the irradiated area also suffer. Various types of cell death occur, including apoptosis, necrosis, pyroptosis, autophagy-dependent cell death, immunogenic cell death, and ferroptosis. Ferroptosis, driven by iron-dependent lipid peroxide accumulation, has been recognized as crucial in radiation therapy’s therapeutic effects and complications, with extensive research across various tissues. This review aims to summarize the pathways involved in radiation-related ferroptosis, findings in different organs, and drugs targeting ferroptosis to mitigate its harmful effects.

## Facts

- Catalyzed by iron and relying on lipid peroxidation, ferroptosis is a type of cell death that has recently been studied in relationship to acute inflammatory and chronic fibrotic damage caused by radiation therapy.
- Ferroptosis contributes to post-radiation cell death across many organ systems, including gastrointestinal, hematopoietic, cutaneous, cardiovascular, ovarian, and neurological.
- Therapeutics aimed at inhibiting several cellular pathways related to ferroptosis have demonstrated preclinical efficacy in preventing and treating tissue-level damage caused by ionizing radiation.

## Open questions

- To what degree does ferroptotic cell death account for the tissue-level changes that characterize fibrotic pathology that follows radiation therapy?
- Does ferroptosis occur following irradiation in all tissue types and how do the pathways that lead to ferroptosis vary between tissue types?
- What translational potential is represented by anti-ferroptotic drugs in preventing or treating fibrotic pathology related to radiation damage, particularly in an oncologic setting?

## Introduction

Ionizing radiation (IR) has played a crucial role in cancer therapy ever since Maria Sklodowska-Curie and Antoine Henri Becquerel discovered its physiological effects in 1901 [1]. Today, approximately 50% of cancer patients undergo radiation treatment [2–6]. However, radiation therapy comes with significant acute and chronic complications, including hypovascularity, poor wound healing, and functional impairment [6].

When ions release energy into cells during irradiation, they cause DNA damage, resulting in base damage and single- or double-strand breaks. Additionally, IR leads to the radiolysis of intra- and extra-cellular water, generating reactive oxygen species (ROS). These ROS damage nucleic acids, lipids, and proteins, triggering various adverse cellular effects, including termination of cell division. Due to the inefficiency of cancer cells in repairing radiation-induced damage, they are more vulnerable to radiation therapy [7]. Nevertheless, all cells in the irradiated field, including healthy cells, suffer damage [8].

Various types of cell death can occur in both diseased and normal tissues, such as apoptosis, necrosis, pyroptosis, autophagy-dependent cell death, immunogenic cell death, and ferroptosis [2, 4, 7]. Traditionally, cell death resulting from IR has been categorized as apoptosis (or Programmed Cell Death (PCD) type I), autophagy (or PCD type II), or necrosis (cell death without PCD type I and II features) [9]. Apoptosis is the most common form of cell death in cancer, characterized by morphological changes like cell shrinkage, nuclear condensation, loss of adhesion to the extra-cellular matrix, and dynamic membrane blebbing [9–12]. However, recent research has revealed that additional types of cell death, such as ferroptosis, play significant roles in the therapeutic effects and complications of radiation therapy.

Ferroptosis is a type of cell death in which iron-dependent lipid peroxide accumulation drives membrane rupture in a non-apoptotic fashion [13]. Since its discovery, investigations into the cellular and molecular mechanisms underlying ferroptosis have yielded further characterization of the process. Ferroptosis has emerged as a significant player in cell death resulting from radiation therapy and has been extensively studied in cancerous, intestinal, hematopoietic, pulmonary, dermal, cardiovascular, ovarian, and neural tissue [8]. As the scientific literature concerning the role of ferroptosis in tissue damage caused by IR has advanced, the objective of this review is to provide a summary of the pathways included in radiation-related ferroptosis, specific findings in different organs, and the drugs that have been explored to target ferroptosis and mitigate its harmful effects.

## The hallmarks of ferroptosis

Ferroptosis is a cell death pathway that has been the focus of increasing investigation due to its theorized role in numerous human diseases. Ferroptosis lacks typical apoptotic features such as chromatin condensation and apoptotic body formation, as well as the formation of autophagosomes, a characteristic associated with autophagy. Instead, ferroptotic cells typically show shrunken mitochondria with increased mitochondrial membrane density and diminished mitochondrial cristae [8].

**Fig. 1:**
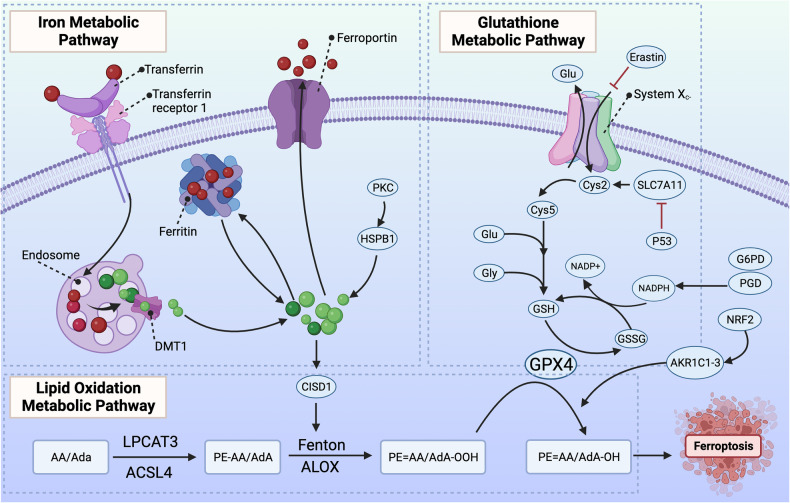
Iron metabolism, GPX4 regulation, and PUFA metabolism are the hallmark pathways involved in ferroptosis.

Ferroptosis is defined by a unique redox imbalance driven primarily through enzymatic or iron-dependent lipid peroxidation and loss of antioxidant membrane repair resulting in membrane rupture and cell death. Excess intracellular free iron favors the generation of ROS via the Fenton reaction [13], which causes oxidative damage to phospholipid membranes and induces ferroptosis. Moreover, polyunsaturated fatty acids (PUFAs) are prone to oxidation by ROS, Lipoxygenase enzymes, cytochrome P450 enzymes, and potentially other oxidative enzymes (Fig. 1: General Ferroptosis Pathways). Thus, excess concentrations of PUFA-containing phospholipids in membranes render them vulnerable to oxidative damage and subsequent ferroptosis [14, 15]. Counteracting this, glutathione peroxidase 4 (GPX4), a phospholipid hydroperoxidase that selectively reduces membrane-bound lipid hydroperoxides to lipid alcohols [16], acts as the primary protective enzyme against ferroptosis [17]. Each of these factors are regulated at the epigenetic to protein level.

### Oxidation of membrane polyunsaturated fatty acids

Lipid peroxidation by ROS and oxidative enzymes is the definitive hallmark of ferroptosis, though the reaction is contingent on the availability of PUFA-containing phospholipids within the membrane. Phospholipids containing at least one PUFA tail are prevalent in mammalian cell membranes [17], where they are essential for maintaining membrane fluidity, membrane protein binding, and membrane protein function [18].

PUFA addition to phospholipids can be regulated by Acyl-CoA synthetase long-chain family member 4 (ACSL4) and lysophosphatidylcholine acyltransferase 3 (LPCAT3). ACSL4 catalyzes the addition of CoA to free arachidonic acid (AA) or adrenic acid (AdA). Subsequently, LPCAT3 catalyzes AA/AdA-CoA inclusion to phosphatidylethanolamine, a common membrane phospholipid [19]. The ACSL4-LPCAT3 pathway therefore increases ferroptosis sensitivity [19–21], though ferroptotic death independent of ACSL4 has also been demonstrated [18]. The strong association between ferroptosis and PUFA processing by ACSL4 and LPCAT3 has led researchers to believe that only membrane-bound and not free PUFAs can initiate ferroptosis [17]. Indeed, Kagan et al. demonstrated that ACSL4 knockdown resulted in higher levels of free oxidized PUFAs, decreased oxidized membrane phospholipids, and reduced cell death [21].

By definition, PUFA tails have one or more bis-allylic carbons, which are essential for ferroptosis execution. ROS interact at this site by abstracting a hydrogen from the central carbon to form a new carbon-centered lipid radical. The carbon radical rearranges and interacts with intracellular oxygen to form a lipid peroxide radical, thus continuing the propagation of free radical generation [14, 22, 23]. Lipoxygenases similarly generate lipid peroxides at the same bis-allylic locus on PUFA tails [23]. Ultimately, ferroptotic cell death likely occurs due to the compositional changes in cell membranes when phospholipids suffer excessive peroxidation, altering ion exchange across the plasma membrane, and causing pores and micelles to form [24, 25]. Lipid peroxides remain highly reactive, and they or their byproducts propagate free radical generation, leading to protein damage and DNA cross-linking [14, 26].

### Generation of ROS by the labile iron pool

Per its name, ferroptosis requires iron, which can catalyze lipid peroxidation. Serum iron is bound by transferrin, enters the cytoplasm via transferrin receptor 1 (TfR1), and is stored as ferritin once internalized [27]. Iron release from ferritin is dependent on lysosomal activity in a process termed ferritinophagy [19].

Following the release, free iron enters the labile iron pool, which is defined as intracellular redox-active iron [28]. Via Fenton chemistry, ferrous iron (Fe^2+^) reacts with hydrogen peroxide to form its oxidized ferric state (Fe^3+^), a hydroxyl anion, and a hydroxyl radical. Subsequently, ferric iron can interact with a second hydrogen peroxide molecule to regenerate its reduced, ferrous state and form a second free radical, hydroperoxyl [14]. Once generated, these ROS interact with PUFA tails of membrane phospholipids, as described previously.

### Loss of lipid peroxide repair

Antioxidant repair by Glutathione Peroxidase 4 (GPX4) represents a crucial protective mechanism against lipid peroxidation and ferroptotic cell death. GPX4 reduces the abundance of damaging lipid peroxides that are responsible for executing ferroptosis [29]. It has been demonstrated that a knockdown of *GPX4* is sufficient to induce ferroptosis, while similarly, upregulated GPX4 activity is able to protect against ferroptosis [30]. GPX4 is the most well-defined membrane repair pathway in ferroptosis defense, though several other important antioxidants are also involved in mitigating lipid peroxidation and ferroptotic cell death, including coenzyme Q_10_ [31, 32], vitamin E [33], and superoxide dismutase (SOD) [34].

## Ferroptosis and ionizing radiation

Several factors, including IR, can disrupt the tightly regulated balance of iron metabolism, lipid metabolism, and antioxidant activity. IR has been found to initiate ferroptosis in both malignant [35] and healthy cells [36], and the ferroptotic pathway has thus received significant attention for its potential anti-tumorigenic effects and involvement with radiation-related collateral injury. Harnessing the potential synergistic effects of ferroptosis-inducers and IR may yield a promising anti-cancer intervention. In contrast, ferroptosis inhibition may offer a means of mitigating the ill effects of radiation-induced injury in healthy tissues.

As an understanding of how IR may induce lipid peroxidation and ferroptosis continues to evolve, several proposed mechanisms have been documented. Importantly, IR can influence ROS generation, iron metabolism, PUFA metabolism, and GPX4 activity.

### IR interacts with PUFA metabolism

IR-induced PUFA peroxidation has been observed as early as 1979, prior to the characterization of ferroptosis [37]. Konings et al. found that sensitivity to lipid peroxidation by radiation is dependent on the membrane lipid composition, with a greater PUFA concentration resulting in greater degrees of lipid peroxidation [37]. Specifically, AA and docosahexaenoic acid were identified as the most vulnerable membrane constituents and are known to be oxidized in cells undergoing ferroptosis [21, 37].

While membrane PUFA concentration has been found to sensitize cells to damage by radiation, IR can similarly induce changes in PUFA metabolism to initiate ferroptosis. IR has been shown to induce the expression of ACSL4 in both cancer cells [35] and healthy tissues [38, 39]. As ACSL4 increases PUFA concentrations in cell membranes [21], ACSL4 upregulation has been hypothesized to contribute to the observed lipid peroxidation, morphological changes, and cell death among irradiated cells [20, 35].

### IR induces iron accumulation and ROS generation

IR increases intracellular iron concentrations and directly generates ROS via the radiolysis of water, which may contribute to the formation of lipid peroxides. IR has been demonstrated to increase iron levels by affecting iron storage [40] and transport [41]. Iron accumulation in tissues has also been partially attributed to radiation-induced hemorrhage [42], a well-established side effect of IR [43]. Additionally, IR has been shown to induce the expression of genes that promote iron uptake while inhibiting genes that encode for iron storage or export [38, 44]. Moreover, Wolszczak and Gaida found that IR can release iron from ferritin [40]. This mechanism is driven by IR-induced Nuclear Receptor Coactivator 4 (NCOA4)-mediated ferritinophagy regulation and has been demonstrated in both intestinal and vascular endothelial tissue, contributing to a cascade of free radical and lipid peroxide formation [44–46].

### IR influences GPX4 expression and cofactor availability

IR can influence GPX4 activity by altering cystine transport, Glutathione Synthetase (GSS) synthesis, and GPX4 expression. IR has been found to reduce SLC7A11 expression [47, 48], a component of system x_c_^-^, the glutamate/cystine antiporter that allows for cystine import that can fuel reduced glutathione (GSH) synthesis [49]. Correspondingly, GSH levels have been shown to decrease in response to IR [38, 41, 45, 47, 50]. Decreased GSH can limit GPX4 function, which requires GSH to restore its reduced, active form [51]. GPX4 expression can also be directly inhibited by IR [39, 44, 45, 48, 50, 52, 53], suggesting a role IR may play in limiting both GPX4 levels and activity. Conversely, however, Lei et al. found *GPX4* and *SLC7A11* expression was upregulated in cancer cells following prolonged exposure to IR [35]. They hypothesized that the expression of ferroptosis-protective genes was an adaptive response to IR therapy, given that the expression levels rose subsequently to IR-induced induction of pro-ferroptotic genes such as ACSL4 [35]. The adaptive response observed by Lei and colleagues may be unique to cancer cells, or the observed differences in gene expression between the studies may be attributable to differences in tissue type or study methodologies. Interestingly, upregulated GPX4 expression has also been observed in keratinocytes following ultraviolet-B (UVB) exposure [54, 55]. These contradictory findings are reflective of the need for further investigation. However, while the effect of IR on GPX4 activity may vary by cell type, it is well established that increased GSH and GPX4 levels are protective against IR-induced ferroptosis in both healthy and malignant cells [35, 51, 56, 57].

## Organ-specific manifestations of IR-induced ferroptosis

Organ systems are understood to be differentially impacted by the adverse effects of IR, with highly replicative tissues – such as epithelial cells or hematological progenitors – being the most vulnerable [58]. However, ferroptosis plays a near-universal role in IR-induced tissue injury (Table 1), which solidifies the importance of investigating ferroptosis inhibition as a radioprotective intervention.

**Table 1:** Ferroptosis induced by IR.

Organ	Pathway	Pathway target	Regulation by IR	Ferroptosis impact	*Source(s)*
Intestine	GPX4	GPX4, GSH	Down	Pro-ferroptotic	Zhang [52] Zhou [44] Ji [39]
			Unchanged	N/A	Wang [107]
	Iron metabolism	NCOA-mediated ferritinophagy, TfR1	Up	Pro-ferroptotic	Zhou [44]
		FPN, FTH, FTL	Down	Pro-ferroptotic	Zhou [44]
		Iron increase; cause unexplored		Pro-ferroptotic	Zhang [52] Wang [107],
	PUFA	ACSL4	Up	Pro-ferroptotic	Ji [39]
			Unchanged	N/A	Wang [107]
		Lipoxygenases (3, 5, 12b, 15)	Up	Pro-ferroptotic	Wang [107] Ji [39]
		LPCAT3	Up	Pro-ferroptotic	Wang [107]
Hematopoietic	GPX4	GPX4	Down	Pro-ferroptotic	Lopez-Nieva [64] Zhang [67] Zhang [42] Yin [66]^a^ Rittase [65]
		GSH	Down	Pro-ferroptotic	Zhang [67]
		SLC7A11	Down	Pro-ferroptotic	Lopez-Nieva [64]
			Up	Protective	Rittase [65]
		SLC3A2	Down	Pro-ferroptotic	Yin [66]^a^
	Iron metabolism	Hemorrhage	Up	Pro-ferroptotic	Zhang [42] Zhang [67] Rittase [65]
		TfR1	Down	Protective	Rittase [65] Yin [66]^a^
		FPN	Up	Protective	Rittase [65] Zhang [42]
		FTH1	Up	Protective	Rittase [65]
			Down	Pro-ferroptotic	Yin [66]^a^
			Unchanged	N/A	Zhang [67]
		IRP1/2	Down	Protective	Zhang [42]
		Iron decrease; cause unexplored		Protective	Li [56]
	PUFA	ACSL4	Down	Protective	Zhang [67] Yin [66]^a^
		15-LOX	Down	Protective	Zhang [67]
Lung	GPX4	GPX4	Down	Pro-ferroptotic	Liu [69] Li [73] Li [70] Li [56] Guo [71]
			Unchanged	N/A	Ji [39]
		GSH	Down	Pro-ferroptotic	Li [56] Li [70]
		SLC7A11	Down	Pro-ferroptotic	Guo [71]
	Iron metabolism	Iron increase; cause unexplored		Pro-ferroptotic	Liu [69] Li [70]
	PUFA	ACSL4	Up	Pro-ferroptotic	Li [56] Liu [69]
			Unchanged	N/A	Guo [71] Ji [39]
		3-LOX, 5-LOX	Up	Pro-ferroptotic	Ji [39]
		15-LOX	Unchanged	N/A	Ji [39]
Skin	GPX4	GPX4, GSH	Down	Pro-ferroptotic	Jiang [38]
		SLC7A11	Unchanged	N/A	Jiang [38]
	Iron metabolism	FTH1	Down	Pro-ferroptotic	Jiang [38]
	PUFA	ACSL4	Up	Pro-ferroptotic	Jiang [38]
Cardiovascular	GPX4	GPX4, GSH, NRF2, SLC7A11, SLC3A2	Down	Pro-ferroptotic	Wu [45]
	Iron metabolism	NCOA4-mediated ferritinophagy	Up	Pro-ferroptotic	Wu [45]
Ovarian	GPX4	GPX4, GSH	Down	Pro-ferroptotic	Zhao [53]
	Iron metabolism	TfR1	Up	Pro-ferroptotic	Zhao [53]
		FPN	Down	Pro-ferroptotic	Zhao [53]
Brain	GPX4	GPX4, SLC7A11	Down	Pro-ferroptotic	Ren [48]
	Iron metabolism	TfR1	Up	Pro-ferroptotic	Ren [48]

*LOX* lipoxygenase, *FPN* ferroportin, *TfR1* transferrin receptor 1, *FTH* ferritin heavy chain, *FTL* ferritin light chain, *SLC7A11* solute carrier family 7 member 11, *SLC3A2* solute carrier family 3 member 2, *GPX4* glutathione peroxidase 4, *GSH* glutathione, *NCOA4* nuclear receptor coactivator 4.

^a^Hormesis response observed by Yin et al. at low doses; effects listed in the table are for radiation doses less than 4.8 gy, while effects were opposite for doses above 4.8 gy [66].

### Gastrointestinal

Radiation-induced intestinal injury (RIII) is a common and significant side effect of abdominal or pelvic radiotherapy [59]. The small intestine is among the most radiosensitive organs in the body [58, 60], though injury can be sustained throughout the GI tract. Understanding novel mechanisms of RIII has been a significant focus of research, and ferroptosis has emerged as a significant contributor. IR can induce ferroptosis in intestinal tissue by increasing ACSL4 expression [39], decreasing GPX4 expression and activity [39, 44] and manipulating iron homeostasis to a pro-ferroptotic state [44, 52].

In intestinal tissues, IR decreases GSH levels and inhibits GPX4 expression, thereby promoting ferroptosis [39]. These findings have been corroborated by several studies in mouse [39, 52] and human intestinal epithelial cells [44]. IR has also been shown to increase iron levels in intestinal tissue through NCOA4-mediated ferritinophagy [44, 52]. Furthermore, PUFA metabolism may play a role in ferroptosis induction by IR through upregulation of ACSL4 in mouse intestinal epithelial cells, and downregulation of this gene was found to be radioprotective in intestinal tissue [39]. Collectively, IR therefore contributes to sensitizing intestinal cell membranes to lipid peroxidation.

The small intestine has a potentially unique relationship with ferroptosis, given its role as the sole site of dietary iron absorption. While iron homeostasis is an important contributor to IR-induced ferroptosis in all cells, few studies have explored the relationship between IR, ferroptosis, and dietary iron absorption and metabolism in the intestine. As iron accumulation favors ROS generation and ferroptosis, it is plausible that the high iron exposure in the intestine is related to the tissue’s increased sensitivity to IR. Indeed, Zhou et al. demonstrated that mice fed with an iron-deficient diet experienced improved survival and significantly mitigated intestinal injury following IR exposure [44]. However, more research is necessary to further characterize this relationship.

The gut microbiome may likewise play an important role in intestinal sensitivity to ferroptosis. Indeed, disrupting the gut microbiome by antibiotic and antifungal therapy has been shown to mitigate effects of ferroptosis by reducing ACSL4 expression in gut tissue [39]. The gut microbiome has also previously been observed to affect colon cancer response to radiotherapy [61], and specific bacteria have been found to enhance radiation injury by down-regulating GPX4 expression [62]. Based on these findings, gut bacteria may thus influence radiation-induced ferroptosis via several mechanisms. RIII remains a debilitating side effect of radiation therapy, leaving a considerable number of patients with acute or chronic symptoms. Furthermore, RIII can limit radiation dosing used to treat cancer. As strategies evolve to shift radiation dose away from intestinal tissue, medications targeting ferroptosis may also prove to be equally important in minimizing the morbidity of gastrointestinal radiation injury.

### Hematopoietic

The hematopoietic system is perhaps the most sensitive organ in the body to the damaging effects of IR. Hematopoietic acute radiation syndrome (h-ARS) is characterized by myelosuppression that occurs at low doses (<3.5 gy of Total Body Irradiation (TBI)), followed by the death of hematopoietic stem cells as the dose increases above 3.5 gy of TBI. The latter is the primary cause of death following exposure to moderate or high doses of IR [63]. While the bone marrow and hematopoietic cells are most affected, other organs involved in hematopoiesis, such as the thymus and spleen, also sustain injury due to IR [64, 65]. Ferroptosis contributes to h-ARS and is initiated by IR-induced iron overload and regulation of ferroptosis-related gene expression [42, 66–68].

IR induces significant increases in the intracellular labile iron pool via viscous hemorrhage. IR causes erythrocytes to extravasate to surrounding tissues and heme-iron is released via macrophage-mediated hemolysis in tissues, which is subsequently degraded to ferrous iron and other constituents [42]. This leads to significant increase in iron deposition in hematopoietic tissues, such as the bone marrow and spleen [42, 65]. Increasing iron levels have been correlated to elevated lipid peroxidation in the spleen [65] and induced ferroptotic cell death in granulocyte-macrophage hematopoietic progenitor cells [42].

IR also induces changes to the expression of other ferroptosis-related genes in hematopoietic tissues. In irradiated mice, IR inhibits *GPX4* [64, 67], *SLC7A11* [64, 65], and also reduced the abundance of the metabolite GSH [67, 68], across several hematopoietic tissues. However, dose-dependent regulation of ferroptosis-related genes may exist. In AHH-1 lymphocytes at low doses (4.8 gy or less), gene expression and protein levels of both pro- and anti-ferroptotic biomarkers decreased. In contrast, expression and protein levels increased to above basal levels from 4.8–18.8 gy and decreased again between 18.8–28.8 gy. ROS levels also varied by IR dose, slightly decreasing at a dose of 4.8 gy or less and increasing significantly at a dose of 7.2 gy and above. Therefore, AHH-1 lymphocytes may demonstrate a hormesis response to IR at low doses [66].

### Lung

Radiation-induced lung injury (RILI) is a prevalent and potentially fatal off-target effect of thoracic radiotherapy, with limited effective interventions available [56, 69, 70]. RILI manifests as alveolar respiratory membrane injury, pulmonary surfactant breakdown, alveolar collapse, inflammatory infiltration, and/or pulmonary edema, which results clinically in progressive dyspnea, compromised lung function, and respiratory failure [35, 70–72]. Radiation-induced pneumonitis (RIP) and radiation-induced lung fibrosis (RILF) are severe subtypes of RILI. RIP and RILF affect 17–50% of irradiated lung cancer patients, resulting in reduced treatment efficacy, elevated risk of mortality and disability, and compromised quality of life [35, 56]. Ferroptosis can contribute to the manifestations of RILI, with IR inducing mitochondrial shrinkage, iron accumulation, ROS, and lipid peroxidation generation, and expression of ferroptosis-related genes [56, 71, 73]. Along with increased iron levels in lung tissue [69, 70], *GPX4*, *SLC7A11*, and *ACSL4* have been the primary biomarkers investigated across studies.

In lungs of irradiated mice, IR downregulates *GPX4* [69–71] and *SCL7A11* [71] and depletes GSH [70]. These findings have been corroborated in human cell models of RILI [56, 71]. Conclusions regarding the regulation of *ACSL4* expression by IR were split across studies. While some reports have shown IR further promotes ferroptosis via the upregulation of the pro-ferroptotic gene *ACSL4* [69], others have not observed a meaningful change in its expression in irradiated human and mice pulmonary epithelial cells, respectively [39, 71]. Nonetheless, ferroptosis in airway epithelial cells may play an important role in driving inflammation and fibrosis, and strategies to minimize ferroptosis may prove to be beneficial in limiting RILI.

### Skin

As many as 85–95% of patients undergoing radiotherapy experience radiation-induced skin injury (RSI) to some degree [74]. RSI can manifest as either acute or chronic pathology, including desquamation and ulceration early and fibrosis or carcinoma late [74, 75]. Ferroptosis is involved in both ionizing and UV RSI. Prior to ferroptosis being described, studies observed significant lipid peroxidation and GSH depletion in the skin of rats exposed to IR [74]. Similarly, activation of anti-ferroptosis pathways has been demonstrated to be protective against RSI [76, 77].

The mechanisms by which IR induces ferroptosis in the skin have recently been clarified: IR may regulate the GSH/GPX4 axis, iron metabolism, and *ACSL4* expression, culminating in increased iron and lipid peroxidation [38]. Jiang et al. observed in human keratinocytes that IR-induced GSH depletion occurred despite unchanged expression of *SLC7A11* and apparent upregulation of Glutamate-Cysteine Ligase, the rate-limiting enzyme responsible for GSH synthesis, suggesting the role of another GSH regulator [38]. They also determined that IR inhibits Ferritin Heavy Chain 1 (FTH1) expression [38], once again implicating the role of iron storage disruption in IR-induced ferroptosis progression. Finally, *ACSL4* expression was elevated in irradiated cells [38]. These findings suggest some similar mechanisms of ferroptosis induction by IR in the skin as in other tissues.

The role of ferroptosis in RSI has garnered greater attention in the context of UV radiation. UV radiation is non-ionizing and does not penetrate the skin but can cause DNA damage and generate ROS [78]. UVB has been shown to induce lipid peroxidation, iron increase, and expression of ferroptosis-related genes in keratinocytes in a similar manner to IR in other tissues [54, 55]. However, studies have also observed that UVB radiation may somewhat conserve antioxidant protection. These findings thus support the role of ferroptosis in RSI, though mechanisms observed following UVB exposure may not be generalizable to IR.

### Cardiovascular system injury

Radiation has been attributed to numerous types of long-term cardiovascular disease, including pericarditis, coronary artery disease, and myocardial fibrosis [79]. The association between low-dose IR exposure and cardiovascular risk remains contested [80–82]. In fact, select studies have recently demonstrated low-dose IR may have therapeutic benefit in select disease states such as heart failure and sarcoidosis [83, 84]. However, it is well understood that in patients exposed to high-dose radiotherapy, the risk of coronary artery disease and cardiac death is significantly elevated [82, 85]. Free radical generation and lipid peroxidation has previously been associated with radiation-induced cardiotoxicity [86, 87], and recently, ferroptosis has been observed to contribute to radiation-induced atherosclerosis progression [45]. In irradiated mouse endothelial cells, Wu et al. observed decreased levels of GPX4, GSH, and NF-E2 related factor 2 (NRF2), which were previously described as protective against ferroptosis. Similarly, IR-induced iron levels to increase, which was attributed to upregulated release from ferritin by NCOA4-mediated ferritinophagy [45]. These studies thus strongly support a role for ferroptosis contributing to cardiovascular disease following radiation therapy.

### Ovarian

Internal reproductive system injury is a significant concern in patients requiring pelvic radiation (e.g., patients with cervical, endometrial, bladder, or rectal cancer), TBI (e.g., patients with hematological malignancies), or cranial/spinal irradiation (e.g., patients with central nervous system tumors) [88, 89]. IR induces ferroptosis in ovarian granulosa cells, associated with elevated iron levels and decreased GSH levels and GPX4 activity [53]. Increased iron levels may be driven by IR-induced TfR1 upregulation and ferroportin downregulation [53]. This evidence indicates the presence of ferroptosis in ovarian IR injury, though further investigation is warranted to fully characterize its role.

### Brain

Brain injury is another severe and common side effect of IR; as many as 50–90% of patients receiving radiotherapy for brain tumors experience disabling cognitive impairment [90]. Ferroptosis has been proposed as one mechanism of neuron injury in response to irradiation and has been observed in hippocampal neurons of irradiated mice [48]. Ren et al. reported that IR induced ROS generation, increased iron levels, and altered expression of ferroptosis-related genes [91]. As identified in several other cell types, radiation was found to affect antioxidant activity via reduced *SLC7A11* and *GPX4* expression while promoting iron import by increasing expression of TfR1 [48]. These studies indicate that ferroptosis occurs in brain IR injury, though the contributions of this pathway are not yet fully understood.

## Current approaches of ferroptosis inhibitors as radioprotective therapeutics

The emerging connections between IR and ferroptosis have generated excitement toward ferroptosis inhibitors as potential interventions to mitigate IR-induced tissue injury. Although the field of ferroptosis remains in its infancy, several promising targets and treatment approaches have already been identified. Given the novelty of this field, the development of precision therapeutics can be challenging, and many of the interventions under investigation require further study to fully understand their mechanisms. Nonetheless, the observed effects on essential downstream biomarkers, such as GPX4, ACSL4, and others, support the efficacy and mechanisms of action of these drugs.

Pharmacological inhibition of ferroptosis can be achieved through two approaches: inducing protective pathways or inhibiting pro-ferroptotic pathways. Protective interventions aim to reduce lipid peroxidation by enhancing GPX4 activity or introducing exogenous lipophilic antioxidants, while inhibitory tactics directly counter IR-induced pro-ferroptotic iron and lipid metabolic pathways. Though most studies have focused on the former approach, interventions targeting the latter mechanism have also shown promising results.

### Activation of the GPX4 pathway is protective against IR-induced ferroptosis

Enhancing GPX4 activity has been the most widely pursued mechanism to prevent tissue injury by IR, successfully mitigating IR-induced ferroptosis in the intestine, liver, lung, ovaries, skin, and CNS tissues (Table 2). Pharmacological upregulation of GPX4 has been achieved by enhancing NRF2 activity, inhibiting GPX4 degradation, and inducing GPX4 expression directly.

**Table 2:** IR-induced ferroptosis inhibitors with a primary mechanism targeting the GPX4 pathway.

Organ	Intervention	Model organism	Primary mechanism	Reference
Intestine	Epigallocatechin-3-gallate (EGCG)	Human (HIEC cells)	Enhances NRF2 activity/ROS scavenge	Xie [93]
	TFERL	Human (HIEC-6 cells)	Enhances NRF2 activity	Wu [45]
	Modified polycysteine peptide	Rat (IEC-6 cells); Mouse	GPX4, GSH upregulation/NOX1 inhibition	Zhang [100]
	Perillaldehyde (PAH)	Human (HIEC-6 cells); Mouse	Enhances NRF2 activity	Tang [95]
Liver	Feulic acid (FA)	Rat	Enhances NRF2 activity	Gawish [94]
Lung	NVP-AUY922	Mouse	Inhibits chaperone-mediated autophagy of GPX4	Li [70]
	Bafilomycin A1 (Baf-A1)	Mouse	Inhibits chaperone-mediated autophagy of GPX4	Li [70]
	PD151746	Human (HULEC-5a cells)	Inhibits PIEZO1-mediated GPX4, SCL7A11 downregulation	Guo [71]
	GsMTx4	Human (HULEC-5a cells)	Inhibits PIEZO1-mediated GPX4, SCL7A11 downregulation	Guo [71]
Skin	LBP	Human (HaCaT keratinocytes)	Enhances NRF2 activity	Jiang [38]
CNS	Melatonin	Mouse (HT-22 hippocampal neurons / in vivo)	Enhances NRF2 activity / ROS scavenge	Ren [48]
Ovary	Sphingosine-1-phosphate (S1P)	Human (KGN cells)	GPX4 upregulation	Zhao [53]

*TFERL* Total flavonoids of *Engelhardia roxburghiana* Wall. leaves, *LBP* *Lycium barbarum* polysaccharide-glycoprotein.

Epigallocatechin-3-gallate (EGCG), total flavonoids of *Engelhardia roxburghiana* leaves (TFERL), *Lycium barbarum* polysaccharide-glycoprotein (LBP), perillaldehyde (PAH), and melatonin are six distinct modalities that upregulate GPX4 expression by enhancing the NRF2 pathway, resulting in amelioration of IR-induced ferroptosis [38, 48, 92–95]. While all six agents increased GPX4 levels, melatonin, LBP, PAH, FA, and EGCG were also found to increase SCL7A11 levels, and TFERL, LBP, PAH, FA, and EGCG also increased levels of GSH [38, 48, 92–95]. Although the effects of NRF2 in ferroptosis are primarily directed at upregulating the GPX4 pathway, NRF2 also plays a role in iron metabolism, namely by promoting iron storage by ferritin [56]. Among these agents, melatonin has received the most attention as a ferroptosis inhibitor in IR-induced tissue injuries. Indeed, melatonin was shown to reduce ROS and increase GSH levels in the colon, liver, ileum, and lung in irradiated rats long before ferroptosis was initially described [48]. In addition to mitigating IR-induced injury, melatonin inhibits ferroptosis in doxorubicin-induced cardiotoxicity, type 2 diabetic osteoporosis, and UVB-induced cataracts, among others [96–98].

Inhibition of GPX4 degradation represents another defense mechanism against IR-induced ferroptosis and tissue injury. Li et al. demonstrated that both bafilomycin A1 (Baf-A1) and NVP-AUY922 conserve GPX4 by inhibiting chaperone-mediated autophagy (CMA). Baf-A1 is a lysosomal inhibitor that has been shown to partially conserve GPX4, GSH, and iron levels in irradiated mice [70]. Baf-A1 inhibits CMA by inhibiting lysosomal acidification and autophagosome-lysosome fusion [99]. Conversely, NVP-AUY922 inhibits HSP90 [70], which disrupts the interactions between core CMA proteins and GPX4, thus preventing GPX4’s degradation and inducing radioprotection.

Finally, GsMTx4 and PD151746 have been found to upregulate GPX4 and SCL7A11 expression, but interestingly this may occur via a pathway not commonly associated with ferroptosis. PIEZO1, a mechanosensitive ion channel, has been found to be an important regulator of IR-induced ferroptosis in pulmonary endothelial cells [71]. Inhibiting PIEZO1 activity directly with GsMTx4 or via calpain, its downstream effector, with PD151746 inhibited ferroptosis in irradiated cells [71]. Both drugs inhibited ROS and lipid peroxide generation by upregulating GPX4 and SLC7A11, partially rescuing cells from the negative effects seen in RILI [71].

Two additional agents, a modified polycysteine molecule and sphingosine-1-phosphate (S1P), have also been found to upregulate GPX4 [53, 100]. GPX4 upregulation, ROS scavenging, and NOX1 inhibition by polycysteine collectively contributed to decreased ROS generation and improved antioxidant protection in response to IR [100]. S1P also exerts radioprotective effects by upregulating the GPX4 pathway, though the precise mechanism in which S1P conserves GPX4 remains not fully understood [53]. However, treatment with S1P significantly reduced vacuole numbers in irradiated cells, suggesting a role of S1P in inhibiting autophagy, which typically promotes ferroptosis via GPX4 degradation [101, 102] and iron release from ferritin [19].

### Introduction of exogenous antioxidants combats ferroptosis

As IR strongly induces ROS generation and inhibits GPX4 antioxidant activity, introducing exogenous antioxidants is an intuitive anti-ferroptosis strategy. The agents described in Table 3 are lipophilic antioxidants that exert their protective effects by scavenging free radicals, thus reducing lipid peroxidation. Ferrostatin-1 (Fer-1) and Liproxstatin-1 (Lip-1) are synthetic antioxidants, and SOD@ARA290-HBc and pMnSOD are modified forms of the endogenous antioxidant SOD.

**Table 3:** Exogenous antioxidants inhibitors of IR-induced ferroptosis.

Organ	Intervention	Model organism	Primary mechanism	Reference
Intestine	Ferrostatin-1 (Fer-1)	Rat (IEC-6 cells)	ROS scavenge/GPX4 upregulation	Wang [105]
	Liproxstatin-1 (Lip-1)	Rat (IEC-6 cells)	ROS scavenge/GPX4, GSH upregulation	Zhang [100]
	Liproxstatin-1	Mouse	ROS scavenge/LPCAT3, 15-LOX inhibition	Wang [107]
Lung	Ferrostatin-1	Mouse	ROS scavenge/GPX4 upregulation	Li [70]
	SOD@ARA290-HBc	Mouse	ROS scavenge/anti-apoptosis / anti-inflammatory	Liu [69]
	Liproxstatin-1	Mouse	ROS scavenge/Enhances NRF2 activity	Li [106]
Hematopoietic	Ferrostatin-1	Mouse	ROS scavenge/GPX4 upregulation	Zhang [67]
Skin	pMnSOD	Human keratinocytes (HaCaT); Rat	ROS scavenge	Wang [77]
CNS	Vitamin E	Mouse (HT-22 hippocampal neurons)	ROS scavenge	Ren [33]

*SOD@ARA290-HBc* superoxide dismutase delivered by bioengineered nanoreactor ARA290-HBc, *pMnSOD* plasmid mitochondrial antioxidant manganese superoxide dismutase.

Fer-1 and Lip-1 were the first highly potent synthetic ferroptosis inhibitors discovered via high-throughput screening and are strong radical-trapping antioxidants [103]. Both compounds have demonstrated efficacy at inhibiting IR-induced ferroptosis in multiple organ systems by direct radical scavenging and downstream effects on the expression of other ferroptosis-related genes in irradiated tissues. In addition to its broad antioxidant effects, Fer-1 has a direct anti-ferroptotic effect by inhibiting the 15-LOX/PEBP1 complex [104] and inducing GPX4 and GSH expression [67, 70, 105]. The effects of Lip-1 have more significant variability than Fer-1 in the literature. Though the antioxidant capacity of Lip-1 is well established [103], researchers have also implicated NRF2 upregulation [106], LPCAT3 and 15-LOX inhibition [107], and GPX4 and GSH upregulation [100] as core mechanisms of radioprotection by Lip-1. Regardless, the role of Fer-1 and Lip-1 in inhibiting IR-induced ferroptosis is widely supported in the literature. Preclinical studies investigating the use of Fer-1 to treat h-ARS, RIII, and RILI all found that the drug imparted protective effects against ferroptosis and IR damage [67, 105, 108].

SOD is an endogenous antioxidant that rapidly reduces superoxide molecules to hydrogen peroxide [69, 109]. Both SOD-dependent agents, SOD@ARA290-HBc and pMnSOD, leverage SOD’s direct antioxidant activity within a bioengineered vessel to improve drug delivery [69, 77]. SOD@ARA290-HBc supplements the effects of SOD with a vector that has anti-inflammatory and anti-apoptotic effects while also improving drug stability and tissue retention [69]. While the predominant effect of SOD is as a ROS-scavenging antioxidant, SOD@ARA290-HBc and pMnSOD both also target other ferroptosis pathways by inducing GPX4 and inhibiting ACSL4 expression [69, 77]. Preclinical support for the potential of these drugs to alleviate IR damage has been shown for SOD@ARA290-HBc in RILI and for pMnSOD in radiation-damaged keratinocytes, demonstrating the translational potential for both of these antioxidant therapeutics [69, 77].

### Disrupting the labile iron pool can inhibit ferroptosis induction by IR

Inhibiting intracellular iron accumulation may also mitigate IR-induced tissue injury due to ferroptosis (Table 4). Fenton-generated ROS due to iron accumulation is a hallmark of ferroptosis and is induced by IR. Deferoxamine (DFO) is an FDA-approved iron chelator that binds non-transferrin-bound free iron [110–112]. DFO has thus received attention as a ferroptosis inhibitor and has been demonstrated to inhibit IR-induced ferroptosis in mouse intestine, human intestinal cells, and human ovarian granulosa cells. In these irradiated tissues, DFO inhibits iron accumulation, which decreases lipid peroxidation and improves cell survival [44, 53]. In addition to direct chelation, DFO also regulates iron metabolic genes. In irradiated human granulosa cells, DFO prevented IR-induced ferroportin downregulation and transferrin receptor (TfR1) upregulation [53]. DFO has also been shown to influence antioxidant pathways, increasing levels of GSH, GPX4, and other antioxidant enzymes [44, 53].

**Table 4:** Drugs with a primary mechanism targeting iron metabolism.

Organ	Intervention	Model organism	Primary mechanism	Reference
Intestine	3-methyladenine (3-MA)	Human (HIEC-6 cells)	Inhibits ferritinophagy	Zhou [44]
Intestine	Deferoxamine (DFO)	Human (HIEC-6 cells); Mouse	Iron chelation/GPX4, GSH upregulation	Zhou [44]
Ovary	Deferoxamine (DFO)	Human (KGN cells)	Iron chelation/GPX4, GSH upregulation	Zhao [53]

The autophagy inhibitor 3-methyladenine (3-MA) has been investigated as another means to inhibit iron accumulation and subsequent lipid peroxidation in irradiated human intestinal epithelial cells [44]. 3-MA has been shown to inhibit cellular iron uptake and promote iron storage by down-regulating TfR1 levels and increasing ferritin heavy and light chain levels, respectively. It was also found to increase GSH levels [44]. These findings suggest that 3-MA may be a promising therapeutic target for preventing radiation injury.

### Targeting PUFA-related pathways inhibits ferroptosis

Finally, inhibition of PUFA synthetic pathways may be protective against IR-induced tissue injury (Table 5). Troglitazone is an antihyperglycemic agent that is also a potent ACSL4 inhibitor. Inhibiting ACSL4 disrupts PUFA metabolism by inhibiting the formation of AA/AdA-CoA from AA/AdA, thus limiting the integration of PUFAs into membrane phospholipids [19]. Treatment with troglitazone was shown to significantly reduce lipid peroxidation and mitigate tissue injury in irradiated mouse intestinal tissue [39].

**Table 5:** Drugs with a Primary Mechanism Targeting PUFA Metabolism or Peroxidation.

Organ	Intervention	Model Organism	Primary Mechanism	Reference
Intestine	Troglitazone	Mouse	ACSL4 inhibitor	Ji [39]
Intestine; Hematopoietic	FerroLOXIN-1/2	Mouse	15-LOX inhibitor	Dar [113]
Hematopoietic	Baicalein	Mouse	15-LOX inhibitor	Thermozier [114]

Baicalein, FerroLOXIN-1, and FerroLOXIN-2 inhibit 15-LOX via different mechanisms, though all reduce direct peroxidation of membrane PUFAs and protect against IR-induced ferroptosis [113, 114]. As a monotherapy, baicalein demonstrated superior survival benefit in mice following TBI compared to Fer-1 or Lip-1. Baicalein also normalized bone marrow protein levels within a week of irradiation [114]. In light of this, further investigation with this agent as a radioprotective ferroptosis inhibitor may be warranted, given the favorable survival benefit conferred vs. known ferroptosis inhibitors.

FerroLOXIN-1 and -2 also protect against IR-induced ferroptosis by inhibiting 15-LOX, though the drug exclusively interacts with the 15-LOX/PEBP1 complex, similar to Fer-1. 15-LOX plays a broader role in the biosynthesis of other lipid mediators, while the 15-LOX/PEB1 complex is more specific to the lipid peroxidation pathway involved in ferroptosis. FerroLOXIN-1 and -2 both have been shown to inhibit ferroptotic death in the ileum and bone marrow, improve ileum integrity, and increase overall survival in irradiated mice [113, 115].

## Future directions

The findings discussed in this review support that ferroptosis induction by IR is universal across tissues and is initiated via regulation of each of the hallmarks of ferroptosis. While variation exists between studies, IR induces lipid peroxidation and ferroptotic cell death via ROS generation, iron accumulation, PUFA metabolism, and inhibition of the protective GPX4 pathway. This conclusion does not vary significantly across tissues except for the hematopoietic system, where hematocytes demonstrate a hormesis response [66]. Despite the conservation of these protective mechanisms, however, hematocytes are the most sensitive tissues to IR [63] and ferroptosis clearly contributes to IR-induced hematocyte cell death [42, 66–68].

While the research discussed in this review focuses on the role of ferroptosis in cell death of tissues directly exposed to IR, there is evidence that ferroptosis plays a role in other systemic side effects of radiotherapy. While apoptosis typically does not initiate an inflammatory response, several studies have suggested that ferroptosis is a pro-inflammatory cell death pathway [116–118]. Similarly, the initiation of ferroptotic cell death can spread between neighboring cells [119] and plays a role in the radiation-induced bystander effect, a process where non-irradiated cells experience damage [120, 121]. However, the mechanism of cell death propagation remains unclear. It is plausible that the molecular mediators of ferroptosis discussed in this review can initiate ferroptosis in neighboring, non-irradiated cells, though further research is necessary to understand this phenomenon.

Despite remaining limitations in our understanding of ferroptosis and its interactions with IR, ferroptosis inhibitors are efficacious mitigators of radiation injury. Ferroptosis inhibitors successfully mitigate radiation injury across several animal models and organ systems, demonstrating again the universality of ferroptosis involvement in IR. Radioprotection by ferroptosis inhibition can be achieved by targeting each core pathway we described: PUFA metabolism, ROS generation, and antioxidant protection. Presently, drugs that increase antioxidant protection by upregulating GPX4 or introducing exogenous ROS scavengers represent the most common mechanism of anti-ferroptotic radioprotection. Ferroptosis inhibition is being explored across a wide range of disease areas, and the number of identified ferroptosis inhibitors has grown extensive [27, 122–124].

While the initial success of these agents is encouraging, ferroptosis inhibition may be a double-edged sword in treating radiation injury. As described previously, ferroptosis is a crucial cell death pathway induced by IR in both cancerous and healthy tissues. Overexpression of GPX4 by cancer cells confers ferroptosis resistance, just as pharmacological upregulation of GPX4 protects healthy tissues [125, 126]. Ultimately, ferroptosis inhibitors must not simultaneously be radioprotective and tumorigenic. Several researchers have begun to identify agents that fulfill these criteria. Notably, Wu et al. found that the radioprotective effects of TFERL have inverse effects in cancer cells, inducing toxicity to three distinct types of human colon cancer cells [92]. Similarly, NVP-AUY922 has previously shown anti-tumor effects in non-small cell lung cancer and breast cancer, while serving a protective role against RILI in mice [70, 127, 128]. Though promising, greater research is necessary to elucidate differential pathways induced in cancer and healthy tissues by IR. Alternatively, tailored drug delivery would enable the localization of ferroptosis protection to healthy tissues while preserving IR’s intended tumor-killing effect [111, 112, 129, 130]. Successfully inhibiting ferroptosis exclusively in healthy tissues will be the most important goal for drug development. However, other considerations include understanding the dose- and time-relationships of IR and ferroptosis-related gene expression, exploring what enables a cell to develop an adaptive response against ferroptosis induction, and comparing IR-induced ferroptosis across tissue types in greater detail.

Lastly, our review stresses the importance of ferroptosis in IR-induced tissue injury, though it is not solely responsible for the injurious effects of IR on healthy tissues. However, the relative contributions of each type of cell death are challenging to quantify. Select studies have compared the relative survival advantage of irradiated tissues that have been administered inhibitors of individual forms of cell death. Many have not found a statistically significant difference in survival with ferroptosis inhibitors compared to inhibitors of other forms of cell death [114, 131, 132], while Zhang et al. theorized ferroptosis may be the primary form of cell death responsible for the development of h-ARS [67]. However, ferroptosis is a complex and incompletely understood process, thus monotherapy inhibitors are unlikely to accurately estimate its total burden on irradiated tissues. Similarly, overlap exists between mediators of different cell death pathways, further complicating the ability to identify relative individual contributions [52, 93, 105]. Regardless, the proportional degree by which apoptosis, ferroptosis, pyroptosis, or other processes are responsible for IR-induced cell death remains an intriguing area for future research. Importantly, understanding differences in the relative role of ferroptosis among other forms of cell death in healthy and malignant tissue may elucidate therapeutic methods to improve both the safety and efficacy of radiotherapy.

## Conclusion

Ferroptosis is universally involved in IR-induced injury across several commonly affected organs. Inhibiting each of the hallmarks of ferroptosis may be effective at mitigating organ injury following IR exposure. However, the precise mechanisms of ferroptosis and the role of IR in its induction require further investigation, which will inform future therapeutic development.
